# Rice copine genes *OsBON1* and *OsBON3* function as suppressors of broad‐spectrum disease resistance

**DOI:** 10.1111/pbi.12890

**Published:** 2018-02-25

**Authors:** Xin Yin, Baohong Zou, Xuexue Hong, Mingjun Gao, Weibing Yang, Xiangbin Zhong, Yang He, Peng Kuai, Yonggen Lou, Jirong Huang, Jian Hua, Zuhua He

**Affiliations:** ^1^ National Key Laboratory of Plant Molecular Genetics CAS Center for Excellence in Molecular Plant Sciences Shanghai Institute of Plant Physiology and Ecology Chinese Academy of Sciences Shanghai China; ^2^ University of the Chinese Academy of Sciences Beijing China; ^3^ State Key Laboratory of Crop Genetics and Germplasm Enhancement Nanjing Agricultural University Nanjing China; ^4^ College of Agriculture and Biotechnology Zhejiang University Hangzhou China; ^5^ College of Life and Environmental Sciences Shanghai Normal University Shanghai China; ^6^ Plant Biology Section School of Integrated Plant Science Cornell University Ithaca NY USA

**Keywords:** rice immunity, bacterial blight, blast, sheath blight, growth, trade‐off

## Abstract

Breeding for disease resistance is the most effective strategy to control diseases, particularly with broad‐spectrum disease resistance in many crops. However, knowledge on genes and mechanism of broad‐spectrum resistance and trade‐off between defence and growth in crops is limited. Here, we show that the rice copine genes *OsBON1* and *OsBON3* are critical suppressors of immunity. Both OsBON1 and OsBON3 changed their protein subcellular localization upon pathogen challenge. Knockdown of *OsBON1* and dominant negative mutant of *OsBON3* each enhanced resistance to rice bacterial and fungal pathogens with either hemibiotrophic or necrotrophic lifestyles. The defence activation in *OsBON1* knockdown mutants was associated with reduced growth, both of which were largely suppressed under high temperature. In contrast, overexpression of *OsBON1* or *OsBON3* decreased disease resistance and promoted plant growth. However, neither *OsBON1* nor *OsBON3* could rescue the dwarf phenotype of the Arabidopsis *BON1* knockout mutant, suggesting a divergence of the rice and Arabidopsis copine genes. Our study therefore shows that the rice copine genes play a negative role in regulating disease resistance and their expression level and protein location likely have a large impact on the balance between immunity and agronomic traits.

## Introduction

Nearly half of the world population consumes rice (*Oryza sativa* L.) as the staple food. However, rice grain production and quality are severely threatened by a variety of pathogens, including hemibiotrophic *Xanthomonas oryzae* pv. *oryzae* (*Xoo*) and *Pyricularia oryzae/Magnaporthe oryzae* (*M. oryzae*), and necrotrophic *Rhizoctonia solani* (*R. solani*). These three pathogens respectively cause bacterial leaf blight, fungal blast and sheath blast, three devastating diseases of rice. Broad‐spectrum and durable resistance is an effective strategy to control disease in rice (Deng *et al*., 2017). Recent studies have characterized dozens of genes contributing to disease resistance and provided potential solutions for the control of diseases in rice. However, much remains unknown on the rice immune machinery. In particular, how the dynamic immunity and the trade‐off between defence and growth are controlled remains elusive in the crop that has been extensively domesticated.

Rice has evolved a complex innate immune machinery during its long co‐evolution with pathogens. Recent advances in genetic and genomic studies have greatly contributed to a better understanding of the complexity of the rice–*Xoo* and rice–*M. oryzae* pathosystems (Niño‐Liu *et al*., 2006; Valent and Chumley, 1991; Wilson and Talbot, 2009). It is proposed that the two‐layered innate immunity responses mainly established in *Arabidopsis thaliana* (referred to as Arabidopsis) (Dangl and Jones, 2001; Jones and Dangl, 2006) also work in rice. The first layer of innate immunity is activated upon detection of conserved pathogen‐associated molecular patterns (PAMPs) or microbe‐associated molecular patterns (MAMPs) by cell surface pattern‐recognition receptors (PRRs), resulting in PAMP‐triggered immunity (PTI). The second layer of innate immunity is activated upon recognition of pathogen‐secreted effectors by intracellular receptors, namely disease resistance (R) genes, leading to effector‐triggered immunity (ETI). It has been generally recognized that PTI is conserved in diverse plant species and acts as a major determinant of basal defence against diverse pathogens. In contrast, ETI provides a race‐specific protection against pathogens with a stronger defence reaction than PTI, often accompanied by rapid programmed cell death at the infection site, namely hypersensitive response (Coll *et al*., 2011). A number of PRRs, mainly receptor‐like kinases (RLKs) and receptor‐like proteins (RLPs), are shown to recognize PAMPs from rice pathogens. For instance, the rice RLP CEBiP and RLK CERK1 can recognize fungal chitins and induce defence responses (Kaku *et al*., 2006; Shimizu *et al*., 2010). For ETI in rice, a large number of effectors have been identified from *M. oryzae* and *Xoo* and dozens have been characterized in terms of their secretion, virulence and the recognition by rice host (Valent and Khang, 2010). Many R proteins have been identified in rice, particularly those conferring broad‐spectrum disease resistance (Liu *et al*., 2013; Verdier *et al*., 2012). Activation of these R proteins induces robust defence responses usually including reactive oxygen species (ROS) production to effectively limit pathogen growth. Interestingly, a pair of antagonistic NLR receptors PigmR and PigmS were epigenetically regulated to fine‐tune durable and broad‐spectrum blast resistance and yield phenotypes in rice (Deng *et al*., 2017).

In the model plant Arabidopsis, the calcium‐binding proteins BON1, BON2 and BON3 are shown to be negative regulators of plant disease resistance (Yang and Hua, 2004; Yang *et al*., 2006). They belong to the evolutionarily conserved copine proteins found in protozoa, plants, nematodes and mammals (Creutz *et al*., 1998). Copine proteins have two calcium‐dependent phospholipid‐binding C2 domains at their amino (N)‐terminus and a putative protein–protein interaction vWA (von Willebrand A) at their carboxyl (C)‐terminus (Rizo and Sudhof, 1998; Whittaker and Hynes, 2002). The BON1 protein resides on the plasma membrane, and this is mainly through myristoylation at its second residue glycine (Hua *et al*., 2001; Li *et al*., 2010). It has conserved aspartate (Asp) residues important for calcium binding in the two C2 domains. These Asp residues are essential for BON1 function, suggesting a regulation by calcium of BON1 (Li *et al*., 2010). The loss‐of‐function mutant *bon1‐1* in Col‐0 accession has an enhanced disease resistance to virulent bacterial pathogen *Pseudomonas syringae* pv. *tomato* (*Pst*) DC3000 and oomycete pathogen *Hyaloperonospora parasitica* (Yang and Hua, 2004). This enhanced resistance largely results from an up‐regulation of the plant immune receptor NLR gene *SNC1* in the absence of pathogen infection (Li *et al*., 2007; Yang and Hua, 2004). The autoimmune phenotype of *bon1* exhibited at normal growth temperature 22 °C can be suppressed at relatively high temperature 28 °C, due to the temperature‐sensitive nature of the NLR proteins (Zhu *et al*., 2010). The three members of *BON1* gene family (*BON1*,* BON2* and *BON3*) in Arabidopsis are all suppressors of immunity, and their triple mutants die from heightened defence responses (Yang *et al*., 2006). Intriguingly, the *bon1* mutants did not close stomata in response to calcium, ABA or bacterial pathogen (Gou *et al*., 2015). Stomata are the entry point of many bacterial pathogens, and their closure upon recognition of PAMP signals is one critical defence mechanism (Melotto *et al*., 2006). BON1 is therefore a positive regulator of stomatal closure in Arabidopsis at the pre‐invasion phase, and this function in stomatal closure regulation is independent of its role in regulating NLR gene expression (Gou *et al*., 2015).

Rice also has two BON1 homologous proteins, with OsBON1 closely related to BON1 and OsBON3 to BON3 (Zou *et al*., 2016). The functions of BON1‐like or copine proteins in rice and other monocots remain unknown. Here, we identified OsBON1 and OsBON3 as negative regulators of disease resistance to *Xoo*,* M. oryzae* and *R. solani*. Transcription of *OsBON1* and *OsBON3* is constitutively activated in the rice autoimmunity mutant *ebr1* (*enhanced blight and blast resistance 1*) and inducible by pathogen infection. The enhanced resistance in *OsBON* mutants is associated with a trade‐off in growth. The *OsBON1*‐mediated defence activation and growth trade‐off are largely suppressed by high temperature. Intriguingly, neither *OsBON1* nor *OsBON3* was capable to complement the Arabidopsis *bon1* mutant. Our study reveals a nonredundant or partitioning function of BON/copine proteins in plant innate immunity in Arabidopsis and rice, two model plants that have evolved independently.

## Results

### *OsBON1* and *OsBON3* are induced by *Xoo* infection

**Figure 1:**
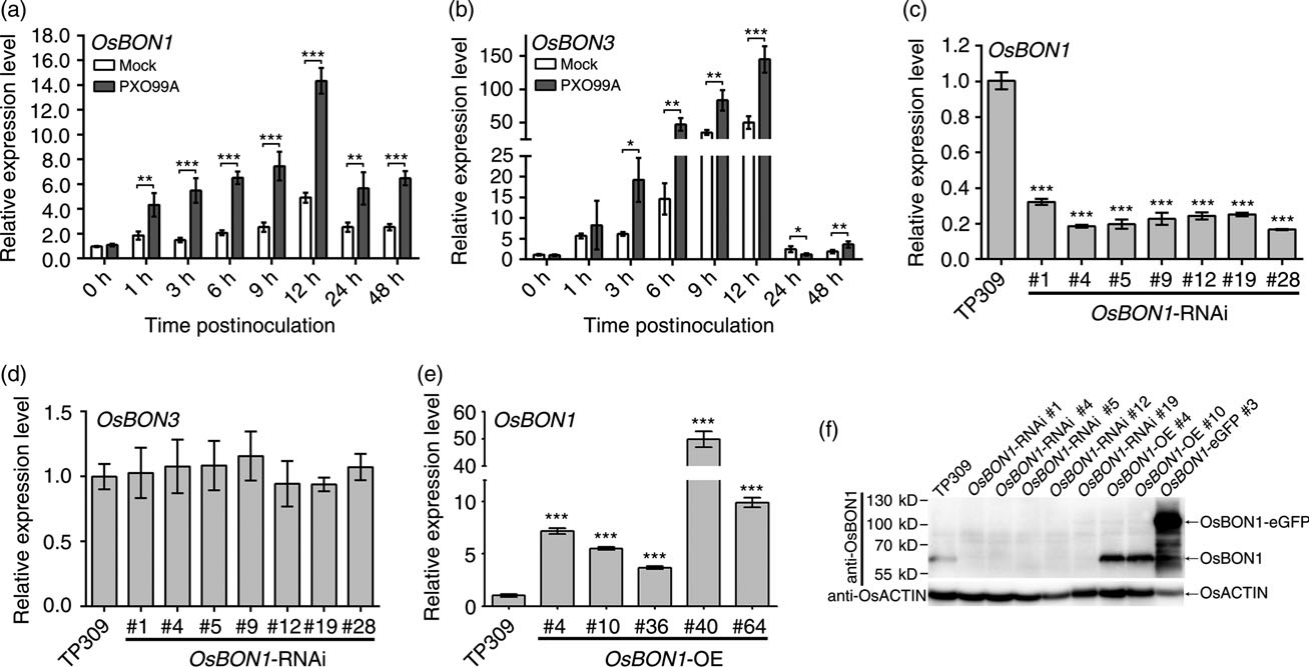
Induction of *OsBON1* and *OsBON3* by *Xoo* and characterization of *OsBON1*‐RNAi and overexpression transgenic plants. (a,b) Induction of *OsBON1* (a) and *OsBON3* (b) RNA expression by *Xoo*. Two‐month‐old plants were inoculated by *Xoo* (strain PXO99A), with mock inoculation (leaf‐clipping with water). Total RNAs were isolated from the inoculated leaves at different time points. Shown are transcript levels of *OsBON1* and *OsBON3* detected by qRT‐PCR. (c–e) RNA expression levels of *OsBON1* (c,e) and *OsBON3* (d) in representative lines of *OsBON1‐* RNAi (c), *OsBON1*‐RNAi (d) and *OsBON1‐OE* (e) compared to the wild‐type TP309 detected by qRT‐PCR. (f) Protein levels of OsBON1 in independent transgenic lines and the wild type detected by Western blot using an anti‐OsBON1 antibody. OsACTIN was used as a control. The rice *OsActin1* gene was used as an internal control to normalize expression levels for qRT‐PCR (a–e). Data are shown as means ± SD from three biological replicates (Student's *t*‐test, *P <* *0.05; **P <* *0.01; ***P <* *0.001) (a–c and e).

In our previous study on the broad‐spectrum disease resistance mutant *ebr1* (Wang *et al*., 2005; You *et al*., 2016), we observed that *OsBON1* (Os02g0521300) and *OsBON3* (Os05g0373300) were up‐regulated in *ebr1* compared with the wild type. Our previous study showed that both *OsBON1* and *OsBON3* were induced by *M. oryzae* (Zou *et al*., 2016). The induction of these two genes was also observed during *Xoo* infection. The expression of both *OsBON1* and *OsBON3* was greatly increased by *Xoo* challenging compared with water mock inoculation (Figure 1a,b). Notably, the relative expression level of *OsBON3* was lower than that of *OsBON1* in all tissue samples (Figure S1a,b). This induction pattern suggests that *OsBON1* and *OsBON3* might play roles in rice immunity.

Quantitative RT‐PCR (qRT‐PCR) analysis showed that *OsBON1* and *OsBON3* transcripts were present in different tissues, with partially overlapping expression patterns. *OsBON1* was more predominantly expressed in leaves (Figure S1a,b). We subsequently generated reporter transgenes using the promoters of *OsBON1* and *OsBON3* to drive β‐glucuronidase (GUS) expression, respectively. GUS staining of transgenic plants of *pOsBON1::GUS* and *pOsBON3::GUS* revealed that both *OsBON1* and *OsBON3* were expressed in the embryo and *OsBON3* was expressed in the coleoptile (Figure S1c,d). At heading stage, *OsBON1* and *OsBON3* expression could be detected in almost all the tissues. High GUS expression was found in young and mature leaves as well as junction of leaf and leaf sheath in *OsBON1::GUS* transgenic lines. High GUS activity was found in junction of leaf and leaf sheath, node region and leaf sheath in *OsBON3::GUS* transgenic lines (Figure S1c,d).

### *OsBON1* negatively regulates rice resistance to bacterial pathogen *Xoo*

To determine the function of *OsBON1* in rice immunity, we obtained transgenic lines with either reduced or increased expression of *OsBON1* in TP309 background. The reduced expression was achieved by RNA interference (RNAi), and the lower expression of *OsBON1* but not *OsBON3* in *OsBON1*‐RNAi lines was verified by qRT‐PCR (Figure 1c,d). The overexpression was achieved by expressing either *OsBON1* or the *OsBON1‐eGFP* fusion gene under the promoter of the maize *Ubi* promoter. The RNA expression of *OsBON1* in *OsBON1‐*OE lines was 5‐ to 50‐fold that of the wild type as analysed by qRT‐PCR (Figure 1e). To further characterize the OsBON1 protein levels in transgenic lines, we performed Western blot using antibodies against OsBON1 (Figure 1f). OsBON1 protein could not be detected in the RNAi lines while it was five‐ to eightfold more in the *OsBON1‐*OE lines compared to the wild type. The *OsBON1*‐eGFP lines also had a higher expression of OsBON1 protein with over 20‐fold increase compared to the wild type (Figure 1f).

**Figure 2:**
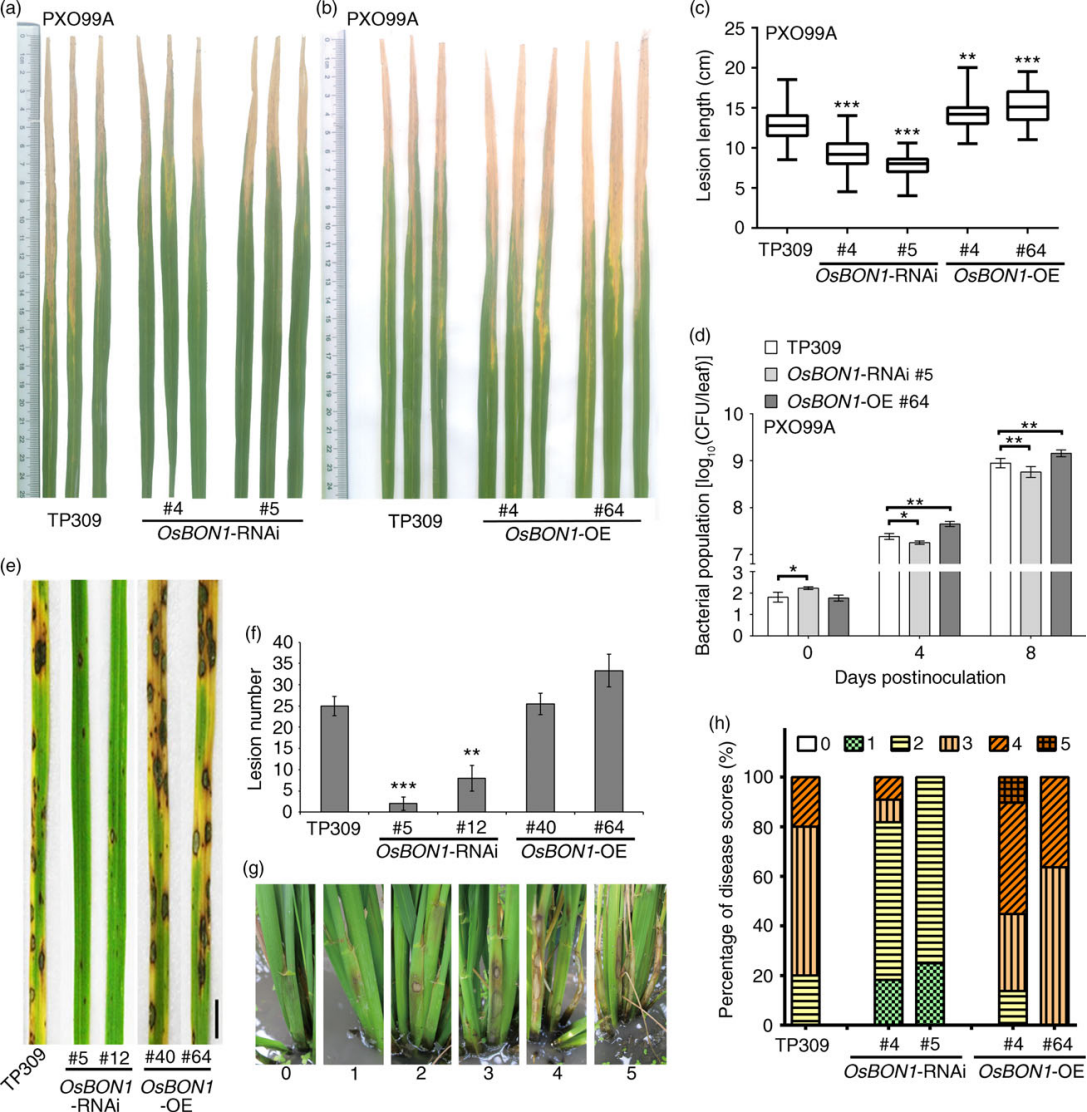
*OsBON1* negatively regulates disease resistance to *Xoo* and fungal pathogens *M. oryzae* and *R. solani*. Shown are disease resistance phenotypes to *Xoo* (a–d), *M. oryzae* (e,f) and *R. solani* (g,h) in *OsBON1*‐RNAi and *OsBON1*‐OE lines. (a,b)Disease symptoms of *OsBON1‐* RNAi (a) and *OsBON1*‐OE lines compared with wild‐type TP309 at 14 dpi with *Xoo* strain PXO99A. (c) Lesion lengths of *OsBON1‐* RNAi and *OsBON1*‐OE lines compared with wild‐type TP309 at 14 dpi with *Xoo* strain PXO99A. Data are shown as box plots (*n *≥* *50). (d) *Xoo* strain PXO99A bacterial populations per leaf were measured at 0, 4 and 8 dpi. Values are shown as mean ± SD (*n* = 3). The bacterial growth curve experiment was repeated twice with similar results. (e) Disease symptoms of the wild‐type TP309, *OsBON1*‐RNAi lines and *OsBON1*‐OE lines at 7 dpi with *M. oryzae* (isolate Hoku1). Scale bars = 2 cm. (f) Average lesion number per leaf of TP309, *OsBON1*‐RNAi lines and *OsBON1*‐OE lines at 7 dpi with *M. oryzae*. Values are means ± SD (*n *≥* *30). This experiment was repeated independently at least three times with similar results. (g) Disease scores (from 0 to 5) used in sheath blight resistance at 14 dpi with *R. solani* AG1‐IA isolate RH‐9. (h) Disease profiles of sheath blight expressed as percentages of six disease scores in TP309, *OsBON1*‐RNAi and *OsBON1‐* OE lines at 14 dpi. Lesion length (c) was measured independently for at least three generations with similar results. Asterisks indicate statistically significant differences in comparison with the wild‐type control (Student's *t*‐test, *P <* *0.05; **P <* *0.01; ***P <* *0.001). Note that the knockdown and overexpression of *OsBON1* significantly increased and decreased *Xoo* resistance, respectively.

We used two stable RNAi lines (#4 and #5) and two stable overexpression lines (#40 and #64) for disease resistance evaluation. We found that the *OsBON1‐*RNAi lines exhibited an enhanced resistance to the *Xoo* strain PXO99A, while *OsBON1‐*OE lines exhibited an increased susceptibility. *OsBON1*‐RNAi had significantly shorter lesions and *OsBON1*‐OE lines had significantly longer lesions than the wild‐type TP309 (Figure 2a–c). *OsBON1*‐RNAi and *OsBON1*‐OE lines were also inoculated with *Xoo* strains PXO71 and PXO347 and similar disease resistance results was observed as inoculated with the *Xoo* strain PXO99A (Figure S2a–d). Disease resistance was also calculated by bacterial growth in the leaves at 0, 4 and 8 days postinoculation (dpi). The colony‐forming units (cfu) of *Xoo* strain PXO99A was reduced in *OsBON1*‐RNAi and increased in *OsBON1*‐OE plants compared to the wild type (Figure 2d). Similar to *OsBON1*‐OE transgenic lines, *OsBON1*‐eGFP lines were also more susceptible to *Xoo* strain PXO99A (Figure S3a,b). Therefore, *OsBON1* plays a negative role in rice basal disease resistance against the bacterial pathogen.

### *OsBON1* negatively regulates resistance to *M. oryzae*

We then analysed the role of *OsBON1* in resistance to rice blast (*M. oryzae*). The wild‐type TP309 and *OsBON1* transgenic plants were assayed for resistance to virulent *M. oryzae* isolate Hoku1 at seedling stage by spray inoculation. Because seeds produced from glasshouse‐grown plants were limited, we performed blast inoculation using additional RNAi line 12 in addition to line 5. The two representative Os*BON1‐*RNAi lines strongly exhibited enhanced blast resistance at 7 dpi, with 3 and 7 lesions per leaf on average on *OsBON1*‐RNAi #5 and #12, respectively, which was significantly reduced compared to the 25 lesions in the wild‐type TP309 (Figure 2e,f). The overexpression of *OsBON1* also increased disease severity because more typical susceptible lesions developed on the *OsBON1*‐OE plants (Figure 2e,f). We further investigated the progressiveness of invasive hyphal growth in leaf sheath with virulent isolate Hoku1. The progressiveness of invasive hyphal growth of *M. oryzae* can be rated by types/stages I–IV as described previously (Kankanala *et al*., 2007): type I, the initial stage of spore germination, germ tube not yet formed; type II, the spores germinated, forming the appressorium (AP); type III, successful invasion of the host cell and formation of primary hyphae (PH); and type IV, hyphae continuing to invade and forming secondary/branch invasive hyphae (BH). We observed that 48% and 31% of penetration sites were type III and type IV, respectively, inside the epidermal cells of the wild‐type TP309 at 38 hpi (Figure S4a). By contrast, only 34%–35% of penetration sites were type III and 15%‐17% were type IV in the two *OsBON1‐*RNAi lines (Figure S4a). Growth of the infectious hyphae was also analysed by Uvitex 2B staining, and a similar inhibition of the hyphae growth was observed in the *OsBON1‐*RNAi lines (Figure S4b‐f). Therefore, the development of advanced infectious phases of blast fungus was drastically delayed in Os*BON1*‐RNAi plants. Although *OsBON1*‐OE lines were indistinguishable from the wild type in *M. oryzae* lesion numbers and infection stage because the wild‐type TP309 is susceptible to *M. oryzae*, more typical susceptible lesions that developed on the *OsBON1*‐OE plants revealed that *OsBON1*‐OE plants had severer disease symptom than the wild type.

### *OsBON1* negatively regulates resistance to necrotrophic fungal pathogen *R. solani*

To test whether *OsBON1* also conditions resistance against necrotrophic pathogens, we performed inoculation experiments with *R. solani*. In contrast to the hemibiotrophic pathogens *M. oryzae* and *Xoo* that invade living rice cells, *R. solani* kills host cells at very early stages of infection (De Vleesschauwer *et al*., 2013). In nature, *R. solani* survives as sclerotia on plant residues in soil (Jia *et al*., 2013), and therefore, we applied field evaluation to measure resistance against *R. solani* for three generations. Two‐month‐old rice plants grown in the paddy field were inoculated, and the degree of disease severity was scored on sheath at 14 dpi giving a value of 0–5 as described (Park *et al*., 2008). A value of 0 represents no lesion; 1 represents the appearance of water‐soaked lesion; 2 represents the appearance of necrotic lesion; 3 represents less than 50% necrosis on the leaf sheath; 4 represents more than 50% necrosis on the leaf sheath; and 5 represents necrosis across the entire leaf sheath resulting in death (Figure 2g). Based on the scores of two independent lines for each transgene, *OsBON1*‐OE lines were found to be more susceptible and *OsBON1*‐RNAi lines more resistant to *R. solani* than the wild type (Figure 2h). *OsBON1*‐eGFP transgenic lines were also sensitive to *R. solani*, similar to *OsBON1*‐OE lines (Figure S3c). Therefore, *OsBON1* also negatively regulates disease resistance to the necrotrophic fungus *R. solani*.

### *OsBON1* and *OsBON3* do not complement the Arabidopsis *bon1* mutant

The Arabidopsis *BON1* gene is also a negative regulator of disease resistance, and we asked whether the rice *BON* genes could complement the Arabidopsis *bon1* defect. However, unlike the Arabidopsis *BON3* gene that rescued the *bon1* mutant when overexpressed (Yang *et al*., 2006), neither *OsBON1* nor *OsBON3* rescued the Arabidopsis *bon1* mutant phenotype when they were overexpressed (Figure S5a). Transcript levels of *OsBON1* and *OsBON3* were detected in transgenic Arabidopsis (Figure S5b,c). OsBON1 accumulation was also detected in transgenic Arabidopsis plants (Figure S5d). This suggests that *OsBON* genes might have diverged from the Arabidopsis *BON1* gene during evolution, given that some amino acids in the conserved C2 and vWA domains in OsBON proteins are different from their Arabidopsis homologs (Zou *et al*., 2016).

### Loss of *OsBON1* function leads to enhanced defence activation

**Figure 3:**
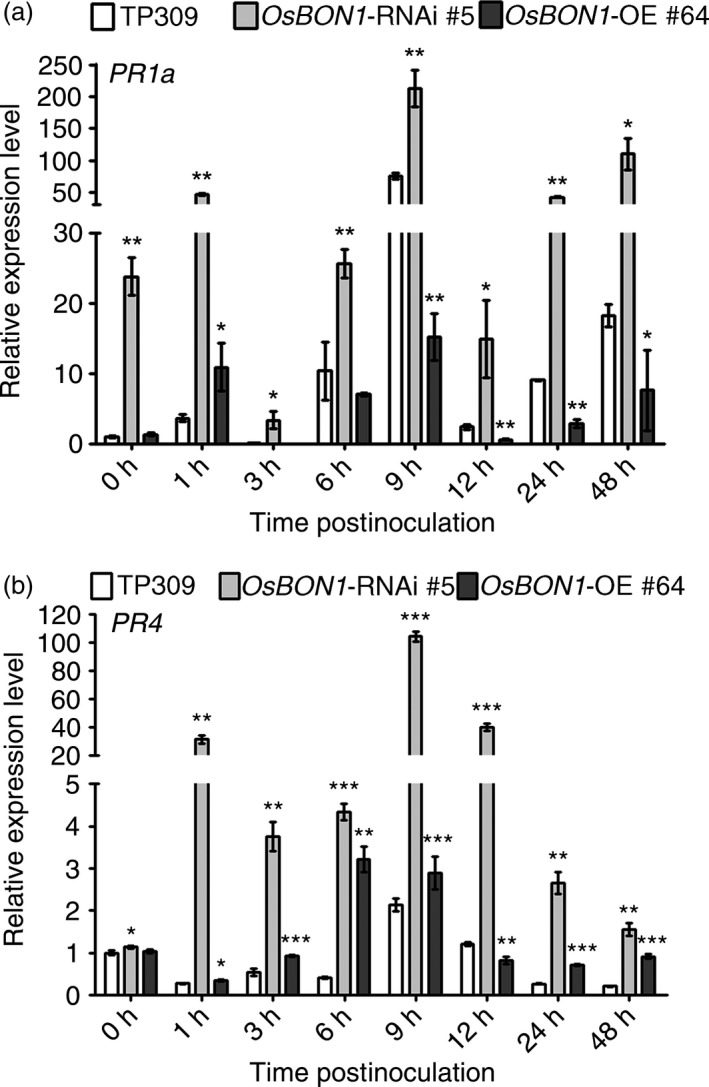
*OsBON1* affects expression of rice defence‐responsive genes. RNA expression of *PR1a* (a) and *PR4* (b) was detected in 2‐month‐old *OsBON1*‐RNAi and *OsBON1*‐OE transgenic and wild‐type TP309 before and after inoculation with *Xoo* PXO99A. The *OsActin1* gene was used as an internal control, and expression levels were normalized to 0 h of TP309. Data are means ± SD (*n *=* *3). Asterisks indicate significant difference in comparison with the wild‐type plants (Student's *t*‐test, *P <* *0.05; **P <* *0.01; ***P <* *0.001).

Activation of plant defence responses during pathogen infection is accompanied by defence hormone pathways, such as salicylic acid (SA) and jasmonate (JA), and up‐regulation of pathogenesis‐related (*PR*) genes (van Loon *et al*., 2006). The *PR* genes *PR1a* and *PR4* are marker genes of SA and JA pathway activation, respectively (Agrawal *et al*., 2000, 2001; Wang *et al*., 2011). We therefore analysed *PR* gene expression in *OsBON1*‐RNAi and *OsBON1*‐overexpressing plants during a time course of 0–48 h in leaves inoculated with *Xoo* (PXO99A). As expected, pathogen challenge strongly induced the expression of *PR1a* in the wild type (Figure 3a). In *OsBON1‐*RNAi plants, *PR1a* was constitutively expressed and was further induced to higher levels than in the wild‐type plants (Figure 3a). *PR4* was not constitutively expressed but was induced by *Xoo* to a higher level in *OsBON1‐*RNAi plants than in the wild type (Figure 3b). In contrast, *OsBON1* overexpression lines had much lower induction of *PR1a* upon *Xoo* challenge (Figure 3a,b). We did not observe significant differences in SA or JA levels among the wild type, *OsBON1‐*RNAi plants and *OsBON1* overexpression plants (Figure S6a‐d). Therefore, the loss of *OsBON1* function likely resulted in enhanced defence activation of both SA and JA responses, but not SA and JA levels because rice has high endogenous SA and JA levels as previously proposed (Yuan *et al*., 2007). A similar synchronous activation of SA and JA was also observed in other rice immunity mutants (Tong *et al*., 2012; You *et al*., 2016).

### *OsBON1* promotes rice growth and development

**Figure 4:**
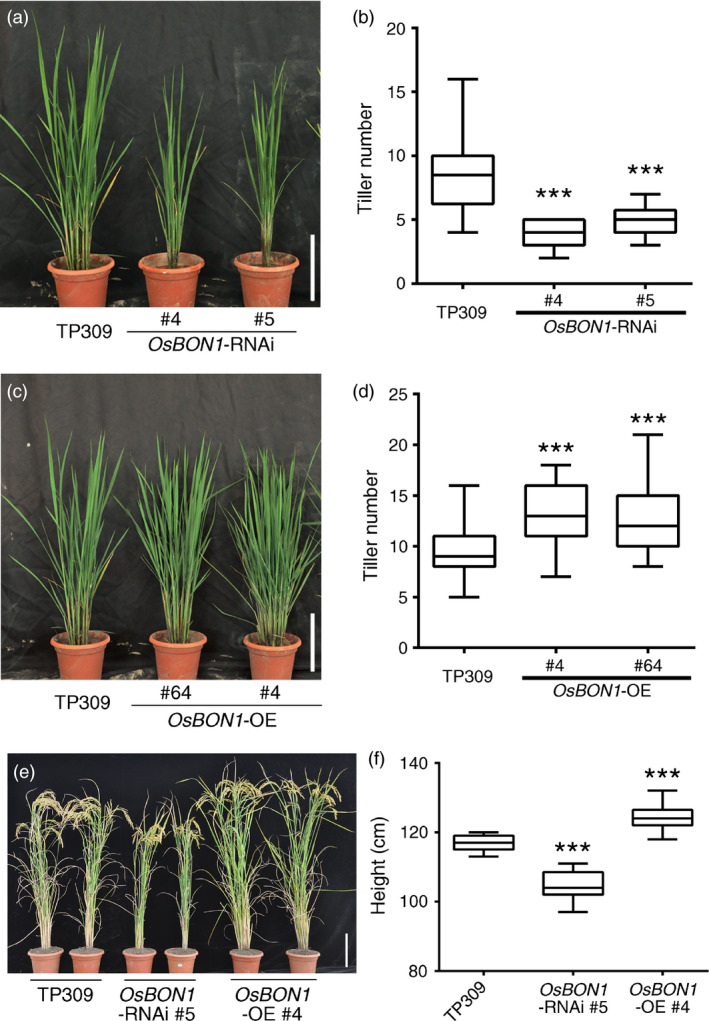
*OsBON1* promotes rice growth and development. (a,b) Morphology (a) and tiller number (b) of 2‐month‐old TP309 and *OsBON1*‐RNAi plants. (c,d) Morphology (c) and tiller number (d) of 2‐month‐old TP309 and *OsBON1*‐OE plants. (e,f) Morphology (e) and plant height (f) of TP309, *OsBON1*‐RNAi and *OsBON1*‐OE mature plants. Data on tiller number and plant height are shown as box plots (*n *≥* *30). Asterisks indicate statistically significant differences in comparison with the wild‐type control (Student's *t*‐test, ***P <* *0.001) (b, d and f). Scale bars = 20 cm (a, c and e).

The transgenic lines of *OsBON1*‐RNAi and *OsBON1*‐*OE* exhibited additional growth phenotypes in tiller number and plant height. At the age of 2 months, compared to the wild‐type TP309, *OsBON1*‐RNAi transgenic lines had fewer tillers (Figure 4a,b), while *OsBON1*‐OE transgenic lines had increased tiller number (Figure 4c,d). At mature stage, *OsBON1*‐RNAi lines had decreased plant height compared to the wild type (Figure 4e,f), while *OsBON1*‐OE lines had increased plant height (Figure 4e,f). These data indicate that *OsBON1* is a positive regulator of plant growth and functions in trade‐off between defence and growth fitness.

### High temperature suppresses the mutant phenotype of *OsBON1*‐RNAi

**Figure 5:**
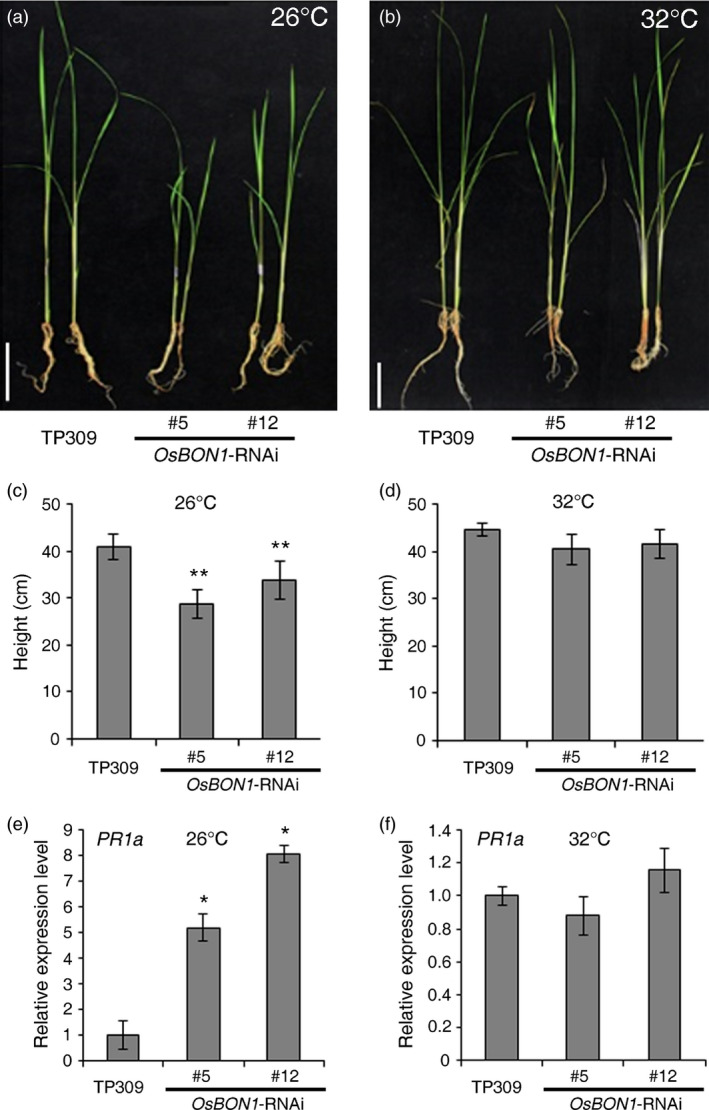
High temperature inhibits the stunted growth phenotype and *PR1a* overexpression of *OsBON1*‐RNAi plants. (a,b) Morphology of 2‐week‐old TP309 and *OsBON1*‐RNAi plants grown at 26 °C (a) and 32 °C (b). Note that the 32 °C growth inhibited the dwarfing of *OsBON* 1‐RNAi plants. Scale bars = 8 cm. (c,d) Plant heights of TP309 and *OsBON1*‐RNAi lines grown at 26 °C (c) and 32 °C (d). Data are means ± SD (*n* ≥ 20). No significant difference was detected at 32° C. (e,f) Expression levels of *OsPR1a* revealed by qRT‐PCR in the wild‐type TP309 and *OsBON1*‐RNAi lines at 26 °C (e) and 32 °C (f). The *OsActin1* gene was used as an internal control. Note that the constitutive activation of *OsPR1a* in the *OsBON1*‐RNAi lines was compromised under high temperature (32 °C). No significant difference was detected at 32 °C. Asterisks indicate statistically significant difference in comparison with the wild‐type control (Student's *t*‐test, *P <* *0.05; **P <* *0.01) (c and e).

In Arabidopsis, the loss‐of‐function *bon1* mutants display a temperature‐dependent defence response (Yang and Hua, 2004), which was attributed to the temperature sensitivity of TIR‐NB‐LRR genes (Zhu *et al*., 2010). Another study also showed that high temperature attenuated the effectiveness of many rice *R* genes against *Xoo*, and *Xoo* was more virulent at high temperature (Webb *et al*., 2010). To test whether the *OsBON1*‐mediated growth and defence activation are also affected by temperature, we compared the growth phenotypes of 2‐week‐old Os*BON1*‐RNAi lines and expression of the defence marker gene *PR1a* in the Os*BON1*‐RNAi lines grown at high temperature (32 °C) and normal growth temperature (26 °C). Indeed, temperature sensitivity was observed in the Os*BON1*‐RNAi lines and in the wild type. The dwarf growth phenotype of 2‐week‐old Os*BON1*‐RNAi plants exhibited at 26 °C was not observed at 32 °C (Figure 5a–d). We further analysed the expression of the *PR1a* genes at two temperatures and found that in contrast to the up‐regulation at 26 °C, the expression levels of *PR1a* in Os*BON1*‐RNAi lines were similar to those of the wild type at 32 °C (Figure 5e,f). This suggests that high temperature likely inhibits the immune activation in the *OsBON1‐RNAi* lines.

### *OsBON3* negatively regulates disease resistance and cell death

**Figure 6:**
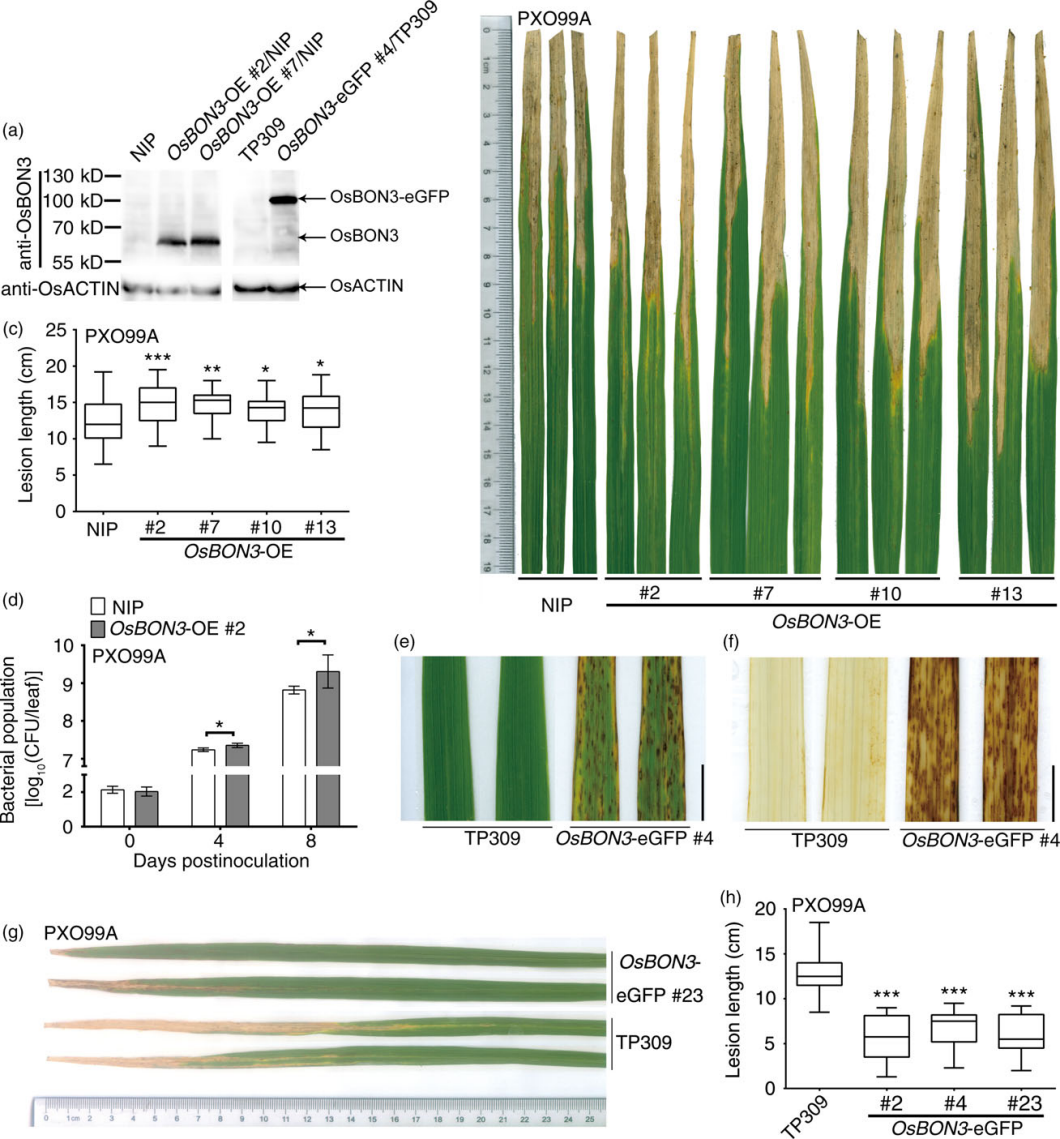
*OsBON3* negatively regulates rice disease resistance. (a) Protein levels of OsBON3 in representative *OsBON3*‐OE and *OsBON3‐eGFP* transgenic lines and wild types detected by Western blot using an anti‐BON3 antibody. (b) Disease symptoms of *OsBON3*‐OE lines in comparison with the wild‐type NIP at 14 dpi with *Xoo* PXO99A. (c) Lesion lengths of *OsBON3*‐OE and NIP at 14 dpi with *Xoo* PXO99A. Data are shown in box plots (*n *≥* *50). (d) Bacterial growth measured at 0, 4 and 8 dpi with *Xoo* PXO99A. Data are means ± SD (*n*  =  3). The bacterial growth curve experiment was repeated twice with similar results. (e) Lesion mimic phenotype of 12‐week‐old *OsBON3‐eGFP* plants. Scale bars = 1 cm. (f) DAB staining of H_2_O_2_ accumulation in twelve‐week‐old *OsBON3‐eGFP* leaves. Scale bars = 1 cm. (g,h) Disease resistance of *OsBON3‐eGFP* lines compared with the wild type at 14 dpi with *Xoo* PXO99A. Shown here are disease symptoms (g) and lesion length (h). Lesion length (h) data are shown as box plots (*n *≥* *50). Lesion length measurement was repeated independently for 3 generations with similar results. Asterisks indicate statistically significant difference in comparison with the wild‐type control (Student's *t*‐test, *P <* *0.05; **P <* *0.01; ***P <* *0.001) (c,d and h).

To determine the function of *OsBON3*, we attempted to generate *OsBON3*‐RNAi transgenic lines but were unable to obtain transformants with decreased expression of *OsBON3*. Instead, we generated *OsBON3* overexpression plants by driving its expression under the maize *Ubi* promoter (*OsBON3‐*OE). Similar to the *OsBON1*‐OE lines, the *OsBON3‐*OE displayed vigorous growth with increased height and more tillers than the wild type (Figure S7a,b). These plants also exhibited a decreased disease resistance to *Xoo* (Figure 6b–d). Therefore, *OsBON3*, similar to *OsBON1*, antagonistically regulates rice disease resistance and growth.

Unexpectedly, overexpression of the *OsBON3‐eGFP* fusion displayed a semidwarf and lower tillering phenotype similar to that of *OsBON1*‐RNAi (Figure S7c). RNA and protein blot assays showed the accumulation of both *OsBON3* transcripts (Figure S7d) and OsBON3‐eGFP protein (Figure 6a) in the *OsBON3*‐eGFP lines. This indicates that they are unlikely RNA‐silenced plants but rather dominant negative lines of *OsBON3*. In addition to the growth defects, the *OsBON3*‐eGFP lines displayed a spontaneous lesion mimic phenotype late at booting stage (Figure 6e). This cell death phenotype was associated with high H_2_O_2_ accumulation in the absence of pathogen (Figure 6f). These *OsBON3*‐eGFP lines also exhibited an enhanced disease resistance to *Xoo* (Figure 6g,h). The enhanced resistance was associated with the development of lesion mimics because the enhanced resistance was not observed at seedling and tillering stages when lesions were not developed. Therefore, *OsBON3*, similar to *OsBON1*, plays a negative role in immunity and promotes growth in rice.

### Subcellular localization of OsBON1 and OsBON3 is affected by pathogen infection

**Figure 7:**
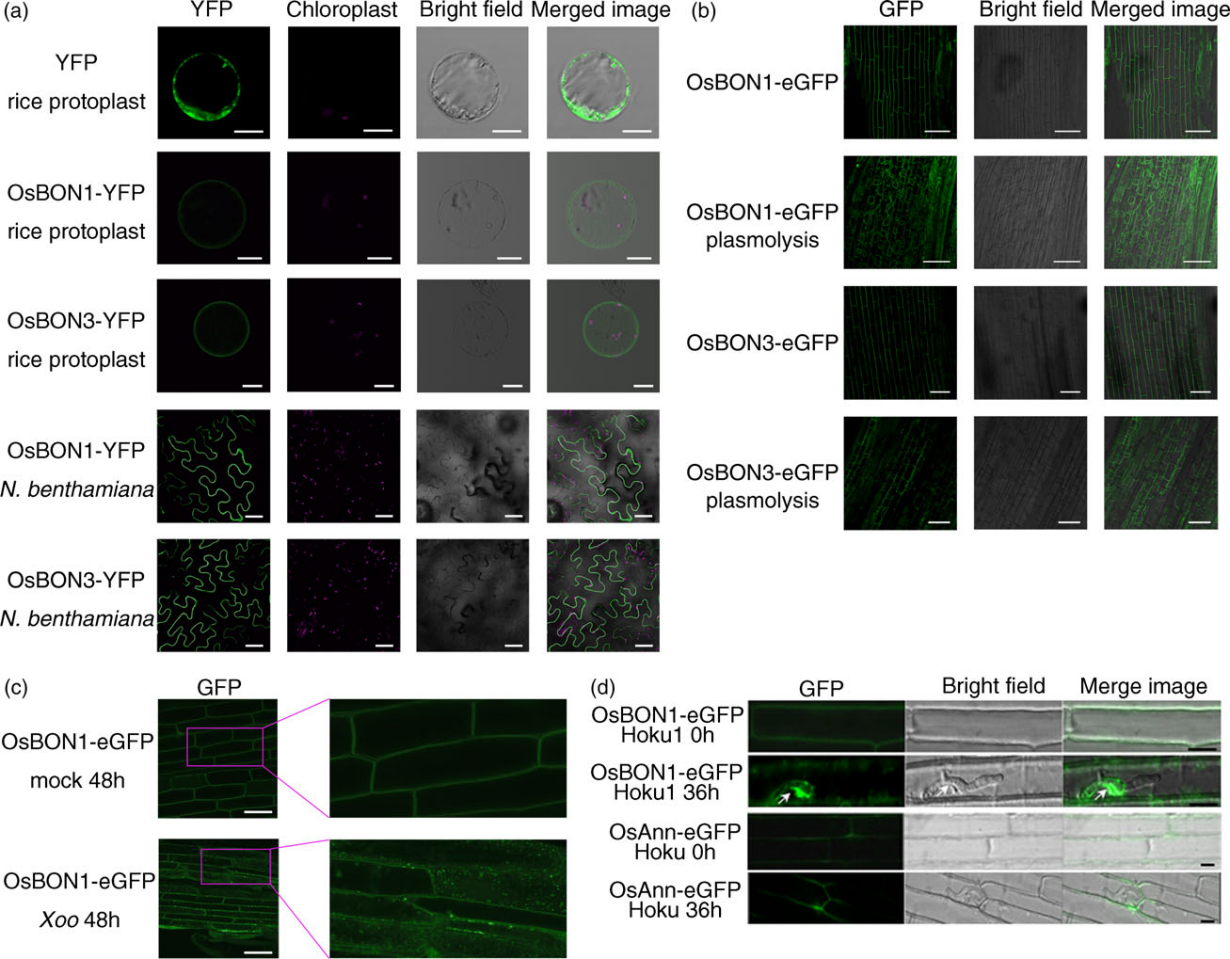
Pathogen infection alters subcellular localization of OsBON1 and OsBON3. (a) Fluorescence signals of OsBON1‐YFP and OsBON3‐YFP expressed in rice protoplasts and *N. benthamiana* leaves. Both proteins are mainly localized to the plasma membrane in contrast to the control protein YFP. Scale bars, 10 μm (rice protoplasts) or 50 μm (tobacco leaves). (b) Fluorescence signals of OsBON1‐YFP and OsBON3‐YFP in root cells of stable transgenic lines of Ubi::OsBON1‐eGFP and Ubi::OsBON3‐eGFP. Plasmolysis was induced using 30% sucrose. Scale bars = 50 μm. (c) Localization of OsBON1‐eGFP protein after infection by *Xoo*. Roots of OsBON1‐eGFP seedlings were infected with *Xoo* or mock‐inoculated with water. Shown are confocal images taken at 48 hpi. Scale bars = 50 μm. (d) Localization change of OsBON1‐eGFP during *M. oryzae* infection. Shown are laser confocal images taken at 0 h (control) and 36 h after leaf sheath inoculation with *M. oryzae*. The transgenic plants with the plasma membrane‐located OsAnn‐eGFP were used as a control. Arrows indicate biotrophic interfacial complex (BIC). Scale bars = 20 μm.

A number of signalling events occur upon pathogen infection at the plasma membrane where the Arabidopsis BON1 is localized. To determine the location of OsBON1 and OsBON3, we generated their YFP fusion proteins OsBON1‐YFP and OsBON3‐YFP driven by the 35S promoter and transiently expressed them in rice protoplasts and *Nicotiana benthamiana* (*N. benthamiana*). The YFP fluorescence signals were concentrated on the plasma membrane in both expression systems (Figure 7a). Stable transgenic lines of the OsBON1‐eGFP and OsBON3‐eGFP were also generated, and the GFP fluorescence signals were also detected on the plasma membrane in root tip cells (Figure 7b). OsBON1 and OsBON3 are predicted to be calcium‐dependent lipid‐binding proteins (Li *et al*., 2010). We therefore determined whether or not the putative calcium‐binding sites are critical for the plasma membrane association of OsBON1 and OsBON3. However, mutating aspartates that are thought to be critical for calcium binding in the OsBON1 or OsBON3 did not alter their locations (Figure S8a–c).

As membrane dynamics is important for plant immunity, we asked whether or not the BON proteins change their localization upon pathogen challenge. We adopted a root inoculation approach (Chen *et al*., 2010) to analyse the dynamics of OsBON localization after *Xoo* infection. In contrast to the sharp and even signals on plasma membrane observed before infection, the OsBON1‐GFP signal became diffused with punctate aggregations both on the plasma membrane and in the cytoplasm after root infection (Figure 7c). A similar internationalization was also observed for OsBON3‐eGFP after root inoculation with *Xoo* (Figure S9). In addition, we used *OsBON1*‐eGFP lines to observe OsBON1 localization after infection with *M. oryzae* using sheath inoculation (Figure 7d). The OsBON1‐eGFP protein was found to relocate to the plasma membrane around fungal haustoria and appeared to be enriched at a special structure named BIC (biotrophic interfacial complex), which is thought to be the secretion site of pathogen effectors (Khang *et al*., 2010). In contrast, the eGFP fusion with a control plasma membrane protein OsAnn‐eGFP did not change the plasma membrane location upon fungal infection (Figure 7d). These results together indicate that pathogen infection triggers relocalization of OsBON1 and OsBON3, which may serve as an early response to pathogen invasion.

## Discussion

Copines are evolutionarily conserved calcium‐binding proteins widely found in protozoa, plants and animals, and their functions have remained largely enigmatic. Our earlier studies have showed that all three Arabidopsis copine genes, *BON1*,* BON2* and *BON3*, are negative regulators of plant immune responses (Yang and Hua, 2004; Yang *et al*., 2006). The loss of *BON1* leads to enhanced resistance to bacterial pathogen *P. syringae* and oomycete pathogen *H. parasitica* in Arabidopsis (Yang and Hua, 2004). The impact of BON1 on immunity is mediated partially through suppressing the expression of the plant immune receptor NB‐LRR genes. Some of these NB‐LRR genes are accession (ecotype)‐specific, which raised questions of whether or not the immunity function observed in Arabidopsis is a conserved function of copine or BON genes of all plants or a unique phenomenon in Arabidopsis. Our current study revealed an important role of rice copine or *BON* genes in immunity. An enhanced and broad‐spectrum disease resistance is found in the RNAi lines of *OsBON1* as well as dominant negative lines of *OsBON3* in rice, suggesting a conserved role of copines as suppressors or negative regulators of immunity in higher plants. It is notable that the rice *OsBON* genes could not rescue the *bon1* mutant phenotype in Arabidopsis. Therefore, copine proteins might have subjected to species‐specific functionality and lost the interexchange capacity in immune responses among higher plants. However, this hypothesis awaits further investigation on additional copine genes in other plants.

Interestingly, knocking down *OsBON* function enhances resistance to both hemibiotrophic (*Xoo* and *M. oryzae*) and necrotrophic (*R. solani*) pathogens in rice. In Arabidopsis, the *bon1* mutant has enhanced resistance to biotrophic and hemibiotrophic pathogens, and its resistance to necrotrophic pathogens has not been tested. It is often assumed that an enhanced resistance to biotrophic pathogens is accompanied by a compromised resistance to necrotrophic pathogens probably due to the antagonistic interaction of SA and JA signalling (Spoel and Dong, 2008). Therefore, it came as a surprise that the *OsBON1*‐RNAi lines exhibit resistance to both hemibiotrophic and necrotrophic fungi. The mechanism of such enhanced resistance is not fully understood. It is evident that knockdown of *OsBON* confers a higher basal expression of defence response genes such as *PR1* and *PR4* and a higher induction of these genes by *Xoo* in rice (Figure 3). However, no significant change was found for the SA or JA levels in the *OsBON* mutants (Figure S6), in contrast to a higher level of SA in the Arabidopsis *bon1* mutant. Our study here suggests that the resistance to both biotrophic and necrotrophic pathogens is at least partially due to the slowing of the fungal invasion in the mutants. In *OsBON1*‐RNAi lines, the development of hyphae was inhibited at early phase (Figure S4). The inhibition was not complete as some hyphae went on to develop further and invade the plants. This could be due to the presence of OsBON3 function or residual function of OsBON1 in the RNAi lines. Further analysis of the double‐knockout mutant will reveal whether the OsBON function is absolutely required for the invasion of fungal pathogens. Nevertheless, the effect on pathogen entry might be a shared mechanism in resistance against biotrophic and necrotrophic fungi in later phase, given that defence‐related genes regulated by both SA and JA signalling are constitutively or more rapidly induced in the *OsBON1*‐RNAi lines (Figure 3), which may provide an explanation of broad‐spectrum disease resistance to both biotrophic and necrotrophic pathogens in *OsBON1*‐RNAi. Similar to OsNPR1 (Li *et al*., 2016), OsBON1 might modify signalling of SA and JA rather than their levels as rice has high basal levels of SA and JA.

This study also reveals a different functional partitioning among members within the copine gene family between Arabidopsis and rice. Like other monocots analysed, rice has two copine genes, with *OsBON1* in clade I and *OsBON3* in clade III. The clade I copine gene in Arabidopsis is duplicated into *BON1* and *BON2*, and like all other dicots analysed, Arabidopsis has one copine gene, *BON3*, in clade III. In Arabidopsis, the loss of *BON1* function leads to autoimmune phenotype, while the loss of *BON2* or *BON3* alone does not lead to obvious immune activation. However, the simultaneous loss of *BON1* and *BON2*, or *BON1* and *BON3* but not *BON2* and *BON3*, leads to stronger autoimmunity than the loss of single *BON1*. This indicates that *BON1* has a major role in immunity regulation, while *BON2* and *BON3* have overlapping functions with *BON1*. In rice, the reduction of *OsBON1* or *OsBON3* dominant negative regulation each leads to enhanced disease resistance. Although the exact contribution of these two rice genes still awaits the analysis of knockout mutants, the lesion mimic phenotype in the *OsBON3* mutant suggests a larger or stronger role of *OsBON3* in immunity and/or hypersensitive cell death than the *OsBON1*. It thus appears that individual copine genes within a species underwent functional partition in modulating immunity, such that Arabidopsis *BON1* assumes a more dominant role while the rice *OsBON1* and *OsBON3* maintain a relatively equal partition. It is likely that clade I and clade III *BON* genes have distinct functions besides overlapping functions, which would also explain their presence in all plant species analysed, and no complementation of *OsBON1* or *OsBON3* with the Arabidopsis *bon1* mutant. The lesion mimic phenotype is seen in the mutant of *OsBON3* but not that of *OsBON1*, also suggesting a distinct function of *OsBON3* if not a higher activity of *OsBON3*.

This study also sheds some lights on the mechanism of how plant copines modulate immune responses. Most strikingly, both OsBON1 and OsBON3 proteins change their localizations upon pathogen invasion. The OsBON1 and OsBON3 proteins form punctate aggregates in the PM and cytosol after *Xoo* infection, while it is concentrated at interface between the host cell and fungal hypha during *M. oryzae* infection. Protein localization changes have been reported for signalling molecules. For instance, the FLS2 and XA21 receptors undergo endocytosis and move from the PM to endosomes (Chen *et al*., 2010; Robatzek *et al*., 2006). Recently, the rice Rac1‐RbohB/H immune complex was reported to move to microdomains upon pathogen invasion (Nagano *et al*., 2016). The immediate change of protein localization indicates that OsBON proteins are also involved in the early event of plant immune responses, either facilitating the signalling to enhance immune responses or antagonizing the signalling to prevent overactivating immune responses. Current evidence could not resolve between the activating and inhibiting models. In the activating model, OsBON proteins might become targets of pathogen effectors and the loss of their functions may be recognized by plants as pathogen invasion signals and thus trigger immune responses. This is consistent with the finding of the involvement of NB‐LRR genes in autoimmunity of the *bon1* mutants that are temperature‐dependent. Whether the enhanced resistance in rice *bon* mutants involves NB‐LRR genes is not known yet, but the temperature sensitivity of the growth defect and *PR* gene expression suggests an involvement of such genes in rice as well. However, the inhibiting model cannot be excluded. The induction of these genes upon pathogen invasion could be a fine‐tuning mechanism in preventing overactivating immune responses in response to pathogens and autoimmunity in the absence of pathogens. Therefore, their loss of function results in higher activation of immune signalling to confer resistance to normally virulent pathogens as observed in the immune homoeostasis controlled by the rice EBR1‐OsBAG4 module (You *et al*., 2016).

A growth‐promoting function of *OsBON1* and *OsBON3* was observed in their overexpression plants. Trade‐off is often observed between growth and defence, and devoting less in immunity could result in more resources for plant growth. Indeed, these overexpression lines exhibited more susceptibility to pathogens in rice. This *OsBON*‐mediated growth phenotype might result from the hormone‐mediated trade‐off between defence and growth in rice (Deng *et al.,* 2017; Yang *et al*., 2013, 2012). However, no significant changes in hormone levels were observed in the RNAi or overexpression lines of *OsBON1* and *OsBON3*. Therefore, the data could support the inhibiting model where *OsBON* genes more directly inhibit immunity. Identifying the direct targets of copine proteins would ultimately differentiate these two models, and the two models may not be mutually exclusive. BON proteins might have different regulatory target proteins in different processes. A recent study of the Arabidopsis BON1 reveals its positive function at stomatal closure control, which could be explained by different target proteins of BON1 in different phases of plant immunity.

The rice *OsBON1* knockdown and *OsBON3* dominant negative lines display broad‐spectrum disease resistance to both bacterial and fungal pathogens. This is a very desirable trait in crop breeding, given that few genes have been molecularly characterized to be involved in such resistance. The identification of *OsBON1/3* in broad‐spectrum resistance will not only enhance a mechanistic understanding of such resistance, but also provide a means to achieve such a resistance. Reduction of copine gene levels could be a potential way to generate resistance to bacterial and fungal pathogens not only in rice but also in other crops due to the likely conservation of copine genes in plants. Finding a right level of reduction of *BON* genes might achieve an enhancement of disease resistance without a substantial yield reduction.

## Experimental procedures

### Pathogen inoculation and disease resistance assay

For *Xoo* resistance assay, 2‐month‐old plants were inoculated with Philippine strain P6 (PXO99A) by the leaf‐clipping method as previously described (Yang *et al*., 2008). Bacteria were incubated on a peptone sucrose agar (PSA) medium at 28 °C for 3 days. Inoculum was prepared by suspending the bacterial mass in sterilized water at a concentration of OD600 = 1.0. Lesion length was measured and recorded 14 dpi. More than 50 leaves per genotype (five leaves per plant) were inoculated for statistical analysis. For bacterial growth curve, 20 cm of leaf tissue infected was ground in 10 mL sterile water to collect bacteria. Bacterial population was counted on PSA plates containing 15 mg/L cephalexin after 3 days of incubation at 28 °C. For *Xoo* induction of genes, plants were infected with *Xoo* strain PXO99A, and RNA was extracted from infected and water mock control leaves collected at different infection time points. Root inoculation was conducted as previously described (Chen *et al*., 2010).

For *M. oryzae* inoculation, virulent isolate Hoku1 was used. Rice seedlings at the three‐leaf stage in a chamber were sprayed with a conidial suspension (5 × 10^4^ conidia/mL) with 0.02% Tween‐20 as described previously (Zou *et al*., 2016). Lesion size and number in the fourth leaf blades of each genotype were scored at 7 dpi with more than 10 leaves per genotype (one leaf per plant). The detailed progressiveness of invasive hyphal growth inside rice sheath cells was conducted and rated by types/stages I–IV as described previously (Kankanala *et al*., 2007). In brief, excised sheaths from 4‐week‐old rice seedlings were cut into 9‐cm strips and inoculated with conidial suspension of isolate Hoku1 (1 × 10^5^ conidial/mL). Inoculated sheaths were incubated in a Petri dish containing wet filter paper such that the conidial suspension settled on the mid‐vein regions. The infectious hyphae in inner leaf sheath cells were observed at 38 hpi under a microscopy. Twenty samples from the inoculated sheaths (10 plants per genotype) were observed, and about 100 infecting hyphae were counted for each genotype.

For *R. solani* resistance assay, *R. solani* AG1‐IA isolate RH‐9 was used for inoculation with tooth picks as described by Wen *et al*. (Wen *et al*., 2015), with some modifications. In brief, sclerotia were transferred to a new PDA (potato dextrose agar) plate and grown for 2 days at 28°C. Short (0.8–1.0 cm) woody toothpicks were sterilized and co‐incubated with fungal plugs for 5 days at 28°C and then inserted into the third leaf sheath of rice plants grown in the paddy field at booting stage. Sheath blight symptom was recorded at 14 dpi with more than 20 sheaths per genotype (two sheaths per plant).

### Other methods

Details of the methods for plant materials and growth conditions, plasmid construction, plant transformation, RNA isolation and gene expression analysis, antibody preparation, protein extraction and Western blotting, SA and JA measurements, histochemical analysis and protein subcellular localization are available in the supplementary methods (Appendix S1). All primers used for plasmid construction and gene expression analysis are listed in Table S1.

## Supporting information


**Figure S1** Expression pattern of *OsBON1* and *OsBON3*.
**Figure S2** *OsBON1* negatively regulates disease resistance to *Xoo* strain PXO71 and PXO347.
**Figure S3** OsBON1‐eGFP transgenic plants displayed enhanced disease susceptibility.
**Figure S4** *M. oryzae* infection stage revealed by Uvitex 2B staining assay.
**Figure S5** *OsBON1* and *OsBON3* do not complement *Atbon1‐1* mutant phenotype.
**Figure S6** *OsBON1* does not affect SA and JA accumulation.
**Figure S7** *OsBON3* promotes rice growth and development.
**Figure S8** Mutations in aspartate residues do not alter the subcellular localization of OsBON1 and OsBON3.
**Figure S9** Subcellular localization change of OsBON3‐eGFP during *Xoo* infection.

**Table S1** Primers used in this study.

**Appendix S1** Supplementary methods.
