# Supplementary material for: Rice copine genes OsBON1 and OsBON3 function as suppressors of broad‐spectrum disease resistance
Source: Plant Biotechnol J. 2018 Feb 25;16(8):1476–87. doi: 10.1111/pbi.12890 (PMC6041448; doi:10.1111/pbi.12890)
Supplement: Supplementary file 1 — Figure S1 Expression pattern of OsBON1 and OsBON3. Figure S2 OsBON1 negatively regulates disease resistance to Xoo strain PXO71 and PXO347. Figure S3 OsBON1‐eGFP transgenic plants displayed enhanced disease susceptibility. Figure S4 M. oryzae infection stage revealed by Uvitex 2B staining assay. Figure S5 OsBON1 and OsBON3 do not complement Atbon1‐1 mutant phenotype. Figure S6 OsBON1 does not affect SA and JA accumulation. Figure S7 OsBON3 promotes rice growth and development. Figure S8 Mutations in aspartate residues do not alter the subcellular localization of OsBON1 and OsBON3. Figure S9 Subcellular localization change of OsBON3‐eGFP during Xoo infection. [file PBI-16-1476-s002.pdf]

Supporting Information

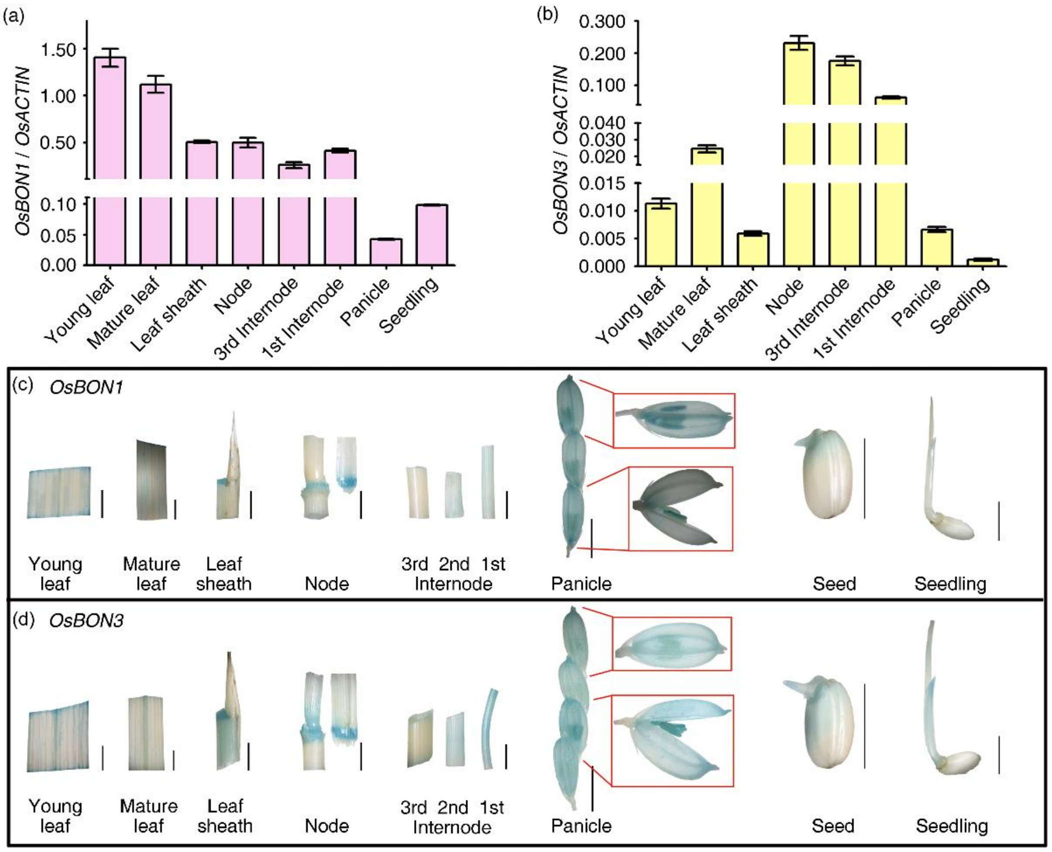

Figure S1. Expression pattern of *OsBON1* and *OsBON3*.

(a, b) RNA expression of *OsBON1* (a) and *OsBON3* (b) in different tissues detected by qRT-PCR. The *OsActin1* gene was used as an internal control. Data were shown as means ± SD (n=3). (c and d) GUS staining of different tissues of *pOsBON1::GUS* (c) and *pOsBON3::GUS* (d) transgenic plants. Scale bars = 5 mm.

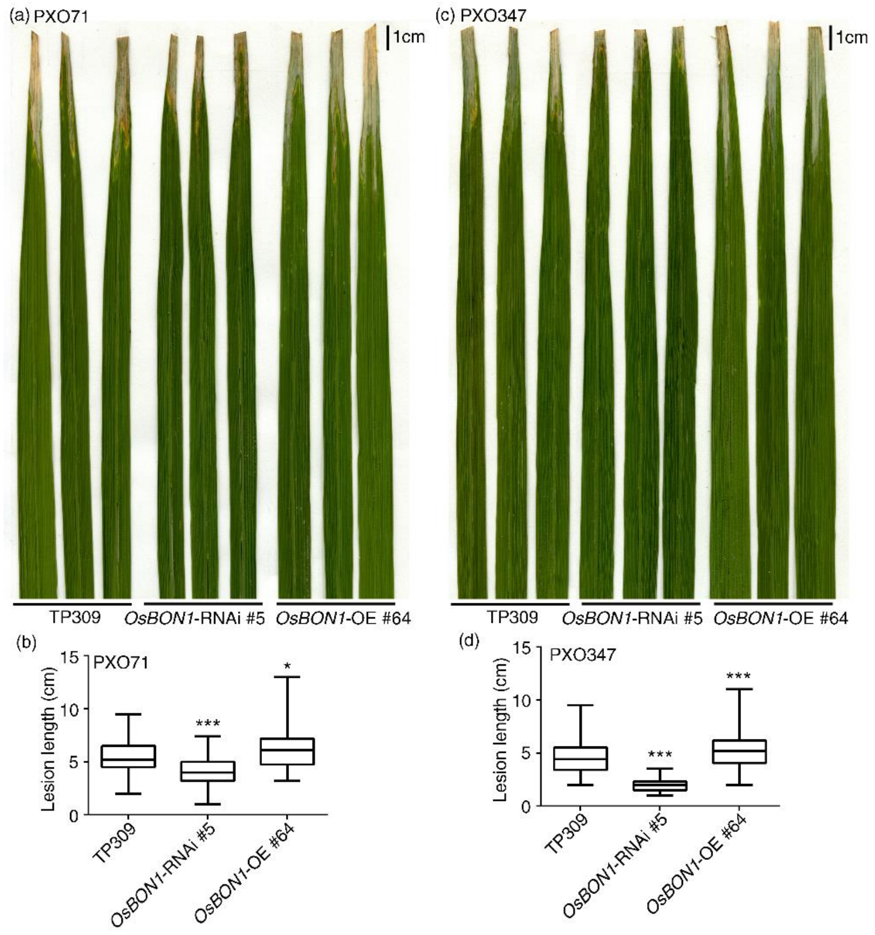

Figure S2. *OsBON1* negatively regulates disease resistance to *Xoo* strain PXO71 and PXO347.

(a, b)Disease symptoms (a) and lesion lengths (b) of *OsBON1*-RNAi and *OsBON1*-OE lines compared with wild-type TP309 at 14 dpi with *Xoo* strain PXO71. Lesion lengths data are shown as box plots (n ≥ 50).(c, d) Disease symptoms (c) and lesion lengths (d) of *OsBON1*-RNAi and *OsBON1*-OE lines compared with wild-type TP309 at 14 dpi with *Xoo* strain PXO347. Lesion lengths data are shown as box plots (n ≥ 50).

Asterisks indicate statistically significant differences in comparison to the wild-type control (Student's *t*-test, *, $P < 0.05$; **, $P < 0.01$; ***, $P < 0.001$).

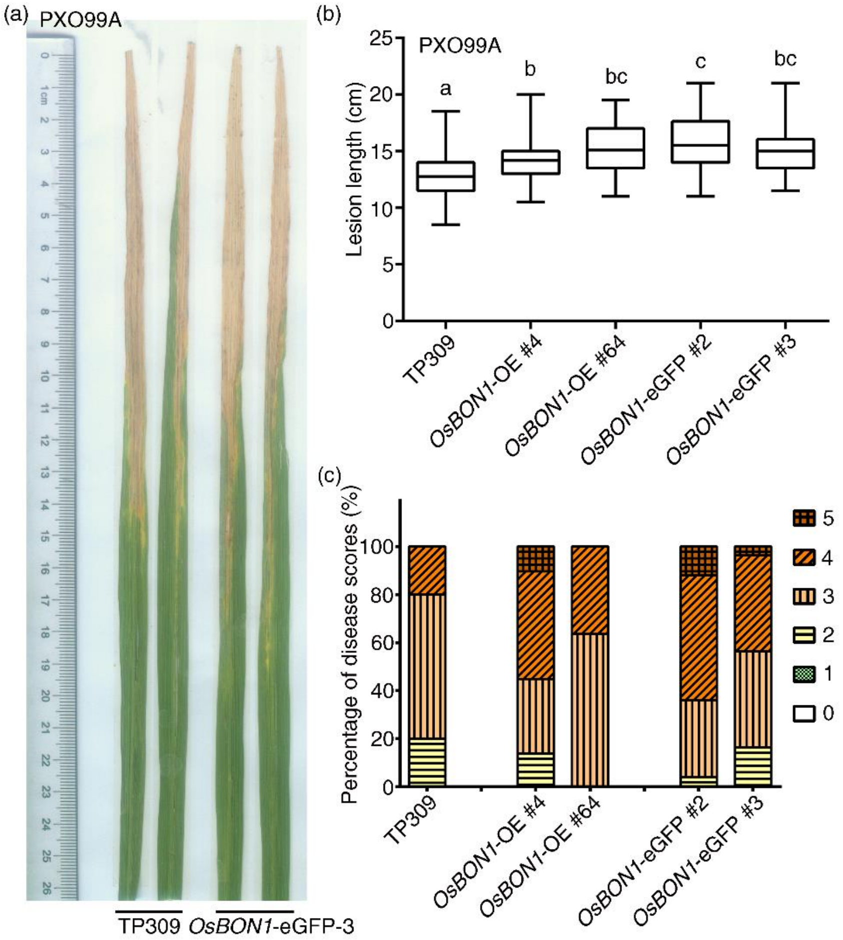

Figure S3. OsBON1-eGFP transgenic plants displayed enhanced disease susceptibility. (a, b) Decreased *Xoo* resistance in OsBON1-eGFP transgenic plants compared to the wild type. Two-month-old *OsBON1*-OE lines and wild-type plants were inoculated with *Xoo* PXO99A, and lesions were measured at 14 dpi. (c) Decrease sheath blight resistance scores in OsBON1-OE and OsBON1-eGFP lines. Disease scores were recorded at 14 dpi with *R. solani* AG1-IA (isolate RH-9). Different letters indicate statistically significant differences (Student's *t*-test and Bonferroni correction for multiple tests, $\alpha = 0.05$).

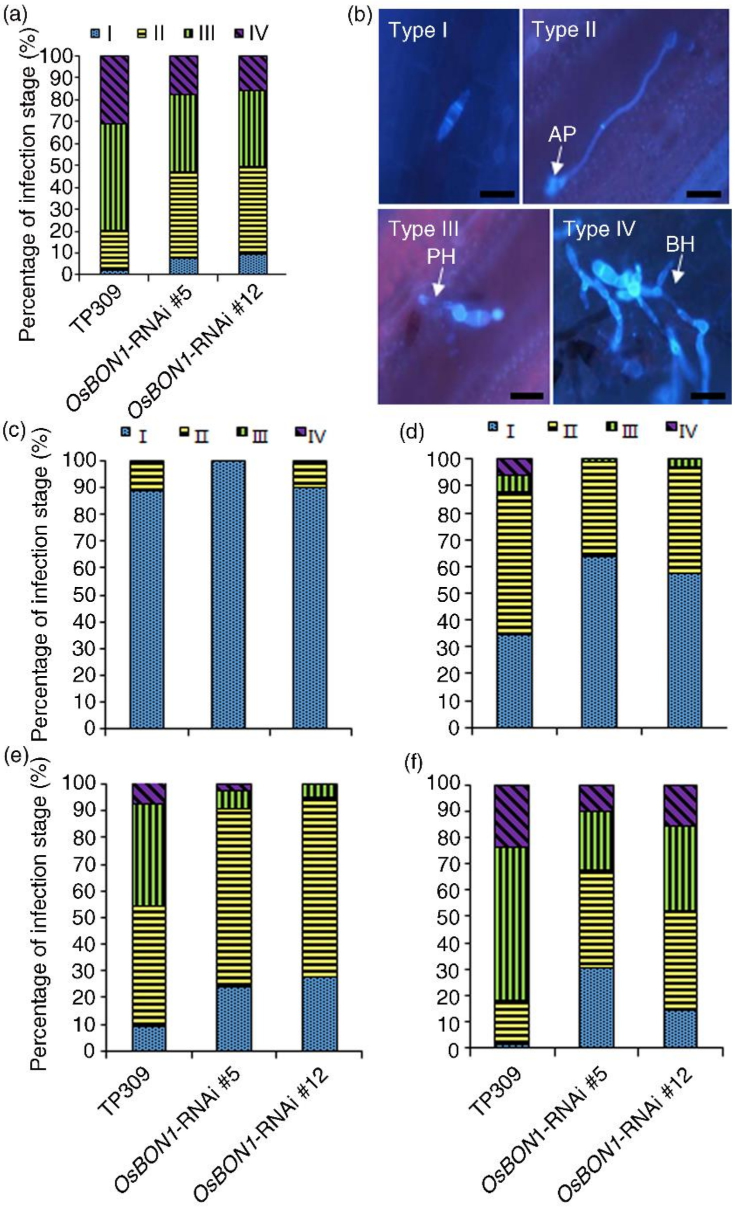

Figure S4. *M. oryzae* infection stage revealed by Uvitex 2B staining assay.

(a) Hyphae infection profiles expressed as percentage of four different types in rice leaf sheaths at 38 hpi with *M. oryzae.* Approximately one hundred infecting hyphae were counted for each genotype and the experiment was repeated three times with similar results. (b) Shown are representatives of the four stages/types of *M. oryzae* invasive hyphae in epidermal cells of the rice leaf sheath. Type I, the initial stage of spore germination, germ tube not yet formed; type II, the spores germinated, forming the

appressorium (AP); type III, successful invasion of the host cell and formation of primary hyphae (PH); type IV, hyphae continuing to invade and forming secondary/branch invasive hyphae (BH). Scale bar = 20 μm. (c-f) Percentages of *M. oryzae* invasive types at 10 hpi (c), 24 hpi (d), 36 hpi (e), and 48 hpi (f) with *M. oryzae* (isolate Hoku1). Fifty hyphae were counted for each genotype and the experiment was repeated three times with similar results.

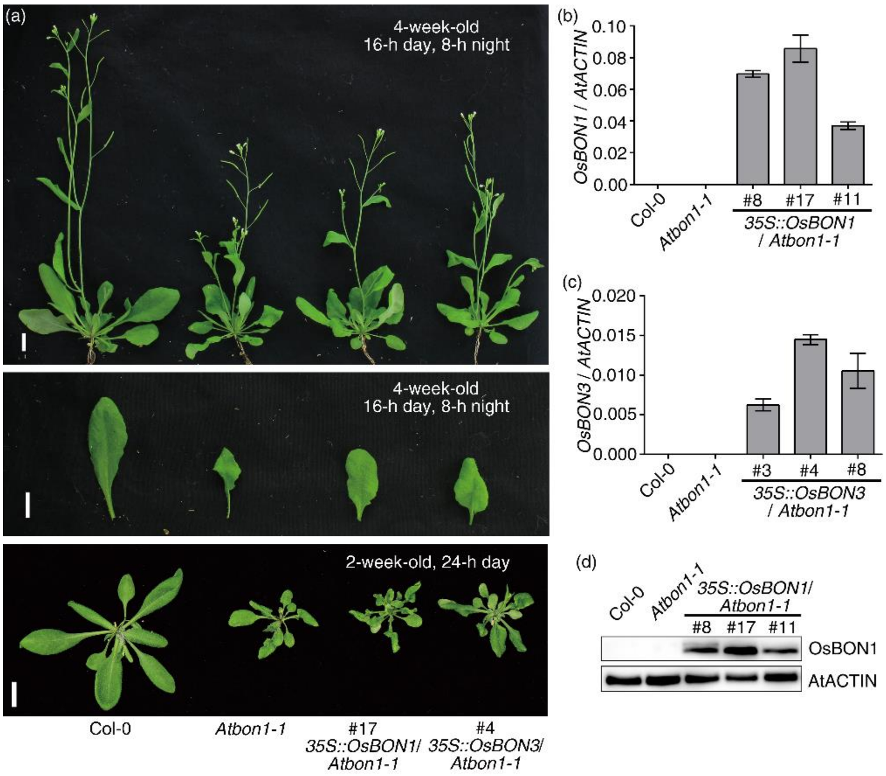

Figure S5. *OsBON1* and *OsBON3* do not complement *Atbon1-1* mutant phenotype.

(a) The *35S::OsBON1* and *35S::OsBON3* transgenic constructs were transformed into the *Atbon1-1* plants. Two-week-old or four-week-old plants were grown under 16 h/day or 24 h/day conditions. Note that neither *OsBON1* nor *OsBON3* could complement the *Atbon1* mutant. Scale bars = 1 cm. (b, c) RNA expression levels of *OsBON1* (b) and *OsBON3* (c) in representative lines of *OsBON1* and *OsBON3* transgenic Arabidopsis compared to the wild-type Col-0 and *Atbon1-1* were detected by qRT-PCR. The Arabidopsis *AtActin* gene was used as an internal control to normalize expression levels for qRT-PCR. Data are shown as means ± SD from three biological replicates. (d) Protein levels of OsBON1 in transgenic Arabidopsis lines and wild-type Col-0 and *Atbon1-1* were detected by Western blot using an anti-OsBON1 antibody. AtACTIN was used as a control.

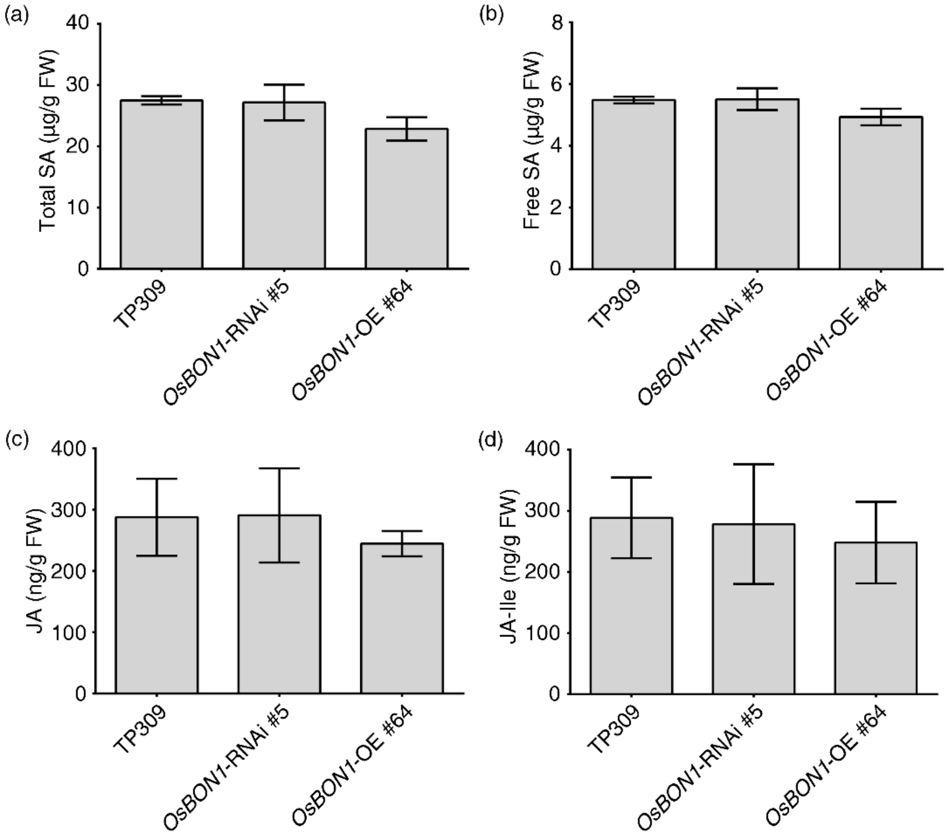

Figure S6. *OsBON1* does not affect SA and JA accumulation.

(a, b) Total SA (a) and free SA (b) in TP309, *OsBON1*-RNAi lines and *OsBON1*-OE lines. Data are means ± SD (n = 3). (c and d) JA (c) and JA-Ile (d) in TP309, *OsBON1*-RNAi lines and *OsBON1*-OE lines. Data are means ± SD (n = 3). No significant difference was detected.

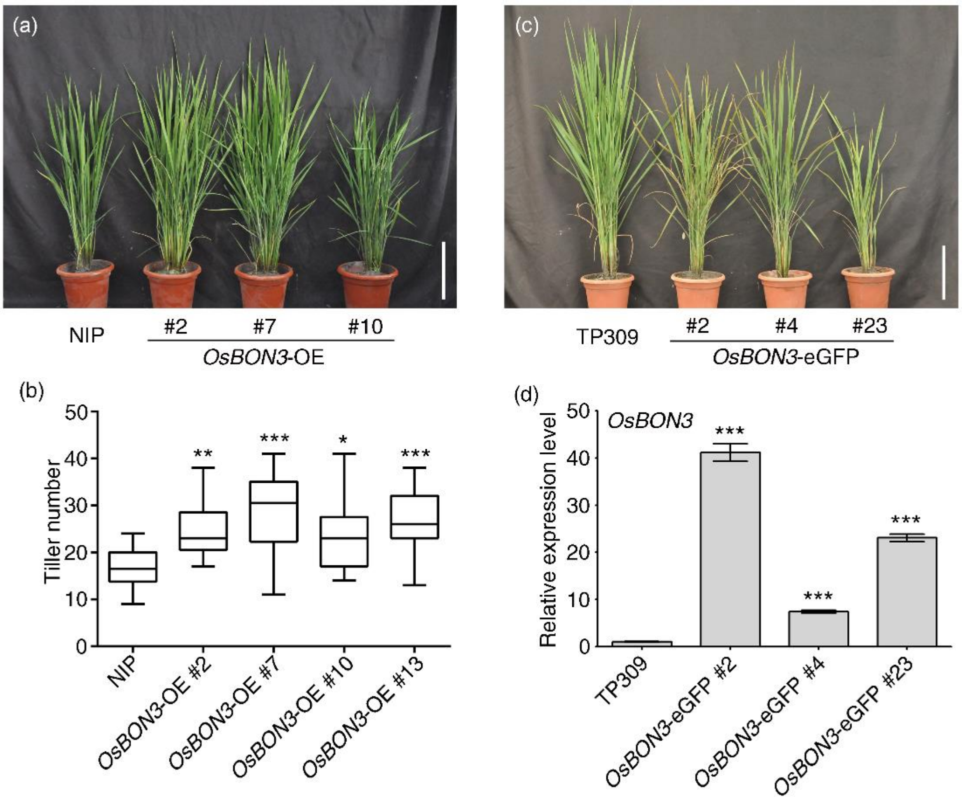

Figure S7. *OsBON3* promotes rice growth and development.

(a) Photographs of two-month-old NIP (wild type) and representative *OsBON3*-OE lines. (b) Tiller number of the wild-type NIP and *OsBON3*-OE lines. Data are shown as box plots (n ≥ 30). (c) Photographs of two-month-old TP309 (wild type) and representative *OsBON3*-eGFP lines. (d) Expression levels of *OsBON3* in 3 independent *OsBON3-eGFP* lines as detected by qRT-PCR. The *OsActin1* gene was used as an internal control. Scale bars = 20 cm (a and c). Asterisks indicate statistically significant differences in comparison to the wild-type control (Student's *t*-test, *, $P < 0.05$; **, $P < 0.01$; ***, $P < 0.001$) (b and d).

(a)

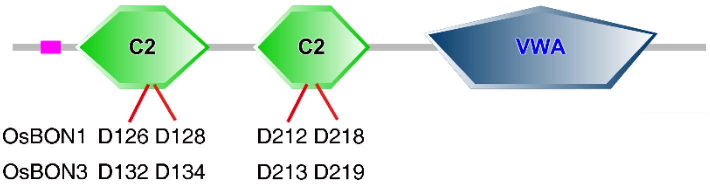

(b)

	OsBON1	OsBON3
M1	D126	D132
M2	D128	D134
M3	D212	D213
M4	D218	D219
M5	D126 D128	D132 D134
M6	D212 D218	D213 D219
M7	D126 D128 D212	D132 D134 D213
M8	D126 D128 D218	D132 D134 D219
M9	D126 D212 D218	D132 D213 D219
M10	D128 D212 D218	D134 D213 D219
M11	D126 D128 D212 D218	D132 D134 D213 D219

(c)

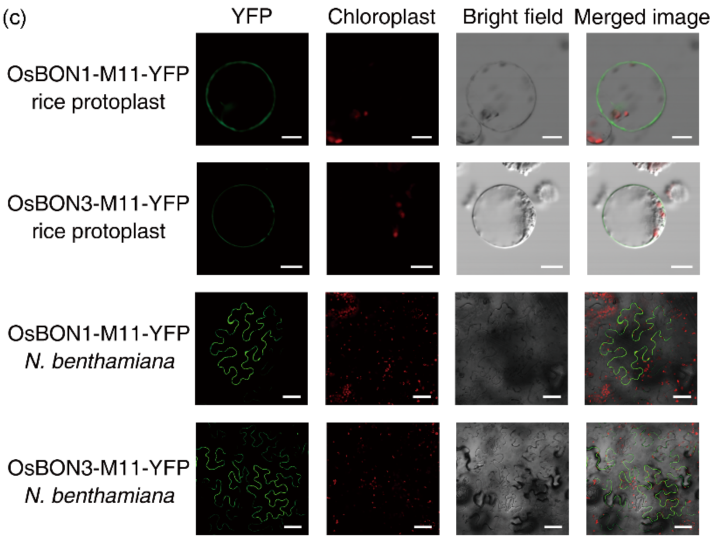

Figure S8. Mutations in aspartate residues do not alter the subcellular localization of OsBON1 and OsBON3.

(a) Diagram of conserved aspartate sites of OsBON1 and OsBON3. The conserved aspartate residues in OsBON1 are Asp^{126} and Asp^{128} in the C2A domain and Asp^{212} and Asp^{218} in the C2B domain, and in OsBON3 are Asp^{132} and Asp^{134} in the C2A domain and Asp^{213} and Asp^{219} in the C2B domain. (b) Combinations of mutations from M1-

M11 in OsBON1 and OsBON3. Aspartates were changed into asparagines singly or in combination. (c) Subcellular localization of OsBON1-M11-YFP and OsBON3-M11-YFP in rice protoplasts and *N. benthamiana* leaves. Scale bars, 10 μm (rice protoplasts) or 50 μm (tobacco leaves).

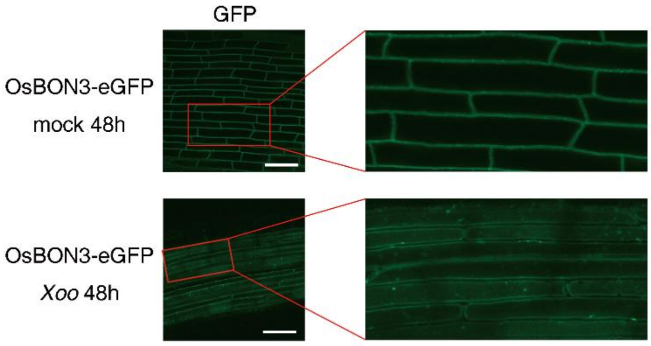

Figure S9. Subcellular localization change of OsBON3-eGFP during *Xoo* infection. Roots of OsBON3-eGFP seedlings were infected by *Xoo*, with water for mock inoculation. Shown are laser confocal images taken at 48 hpi. The cells in the boxes were magnified (right). Scale bars = 50 μm.

Table S1. Primers used in this study.
