# Supplementary material for: Rice copine genes OsBON1 and OsBON3 function as suppressors of broad‐spectrum disease resistance
Source: Plant Biotechnol J. 2018 Feb 25;16(8):1476–87. doi: 10.1111/pbi.12890 (PMC6041448; doi:10.1111/pbi.12890)
Supplement: Supplementary file 3 — Appendix S1 Supplementary methods. [file PBI-16-1476-s001.docx]

**Supplementary methods**

**Plant materials and growth conditions**

Experiments were performed with the wild-type Taipei 309 (TP309) and Nipponbare (NIP) (*Oryza sativa* L. ssp. *japonica*) and related transgenic plants. Plants were grown either in the paddy field under natural growing conditions in the Shanghai and Nanjing (East China, summer crop seasons) or Hainan (South China, winter-spring crop seasons) or in growth chambers under conditions of 12-14h day, 28°C, 80% RH followed by 10-12h night, 26°C, 60% RH.

**Plasmid construction**

For overexpression, the cDNAs of *OsBON1*(1,755 bp) and *OsBON3* (1,791 bp) were amplified by PCR using primers OsBON1-OE-F/R and OsBON3-OE-F/R (Table S1) and inserted into the binary vector pUN1301 to generate the *OsBON1-*OE and *OsBON3-*OE overexpression constructs. For *OsBON1-*RNAi cloning, a short fragment in the conserved sequence of the *OsBON1* coding region was amplified with primers OsBON1-RNAi-BK-F/R and its reverse complement sequence with primers OsBON1-RNAi-SS-F/R (Table S1). The fragments were inserted into vector pTCK303 (Wang *et al.,* 2004).

For GUS fusion reporter constructs, the 3,512-bp or 3,721-bp promoter region of *OsBON1* or *OsBON3* was amplified using primers OsBON1-promoter-F/R and OsBON3-promoter-F/R (Table S1), respectively, and then inserted into the expression vector pCAMBIA1300-GUS-Nos to generate pOsBON1::GUS and pOsBON3::GUS.

For protein localization, the OsBON1-eGFP and OsBON3-eGFP fusions were made by in-frame fusion of the 1,755-bp full-length *OsBON1* (with primers OsBON1-eGFP-F/R) and 1791-bp full-length *OsBON3* (with primers OsBON3-eGFP-F/R) (Table S1) coding sequence with enhanced green fluorescent protein (eGFP). The fusion genes were inserted into the vector pUN1301 to generate the plasmid *Ubi::OsBON1-GFP* and *Ubi::OsBON3-GFP*.

For YFP fusions used in transient expression in rice protoplasts, the coding sequences of *OsBON1* or *OsBON3* were amplified with primers OsBON1-pA7-YFP-F/R or OsBON3-pA7-YFP-F/R (Table S1) and inserted into pA7-YFP, respectively. For YFP fusions used in transient expression in *N. bethamiana*, the coding regions of *OsBON1* or *OsBON3* were amplified with primers OsBON1-1300-YFP-F/R or OsBON3-1300-YFP-F/R (Table S1), and cloned into the vector pCAMBIA1300-N1-35S-YFP, respectively.

Site-directed mutagenesis of OsBON1-M1~M11 and OsBON3-M1~M11 were carried out with appropriate primers listed in Table S1. Mutated genes were inserted into pA7-YFP or pCAMBIA1300-N1-35S-YFP, respectively.

**Plant transformation**

For stable transgenic lines, constructs were transformed into wild-type TP309 or NIP embryogenic callus by *Agrobacterium* (EHA105)-mediated transformation. For transient expression in protoplasts, constructs were transformed into rice protoplasts according to the previously reported method (Yang *et al.,* 2013). For transient expression in *N. benthamiana*, plasmids were transformed into *Agrobacteria* (GV3101) and then infiltrated into *N. benthamiana* leaves together with *p19* (35S::P19-HA) for transient expression.

**RNA isolation and gene expression analysis**

Total RNAs were prepared from rice tissues using TRIzol reagent (Invitrogen) according to the manufacturer’s instructions. For RT-PCR, total RNAs (2 μg) was reverse-transcribed into cDNA using oligo(dT) primer and SuperScript III reverse transcriptase (Invitrogen) and then used as templates for quantitative real-time qRT-PCR with gene-specific primers with *OsActin1* as an internal control. The qRT-PCR experiments were performed using the SYBR Premix Ex Taq kit (Takara) and the Mastercycler realplex detection system (Eppendorf). Three biological were performed for expression analysis. The primers for qRT-PCR are listed in Table S1.

**Antibody preparation, protein extraction and Western blotting**

The coding sequences of OsBON1 (1-335 aa) and OsBON3 (44-330 aa) were cloned into pGEX-4T-1, and used as antigens to raise polyclonal antibodies in rabbit, respectively. Total proteins were extracted from fresh seedlings or leaf tissues. 0.1g tissues were ground in liquid nitrogen and subsequently incubated in 200 μL protein extraction buffer [100mM Tris-HCl, pH 7.5, 300mM NaCl, 2mM EDTA, 10% glycerol, 1% TritonX-100, 1:100 Complete Protease inhibitor cocktail tablets (Roche)] at 4°C for 2 h. Insoluble cell debris were removed by centrifugation at 10,000 rpm for 10 min at 4°C. Proteins were separated on 10% SDS/PAGE gel, and signals were visualized by using ECL systems, and images were captured and analyzed using the Tanon-5200 Chemiluminescent imaging system (Tanon).

**SA and JA measurement**

Preparation and measurement of SA and JA were performed as previously described (Tong *et al.,* 2012). Briefly, 0.1 g leaf tissues from two-month-old plants were ground for SA and JA preparation, with three biological replicates of each genotype. Contents of JA and SA were determined by GC-MS using labeled internal standards.

**Histochemical analysis**

Accumulation of hydrogen peroxide (H_2_O_2_) was detected by 3,3’-diaminobenzidine (DAB) staining as described previously (Ning *et al.,* 2004) with minor modifications. Briefly, leaf tissues were vacuum-infiltrated in DAB solution [1mg/mL DAB, 10 mM MES, pH 3.8 with 0.2% (v/v) Tween-20] for 5 min and incubated at 25°C for 8 h in the light. Samples were then cleared by boiling in 96% ethanol for 10 min. The cleared samples were mounted in 50% glycerol for photography.

For GUS staining, different tissues and organs were collected and incubated at 37°C in GUS staining solution: 50 mM NaPO_4_ buffer (pH 7.0), 5 mM K_3_Fe(CN)_6_, 5 mM K_4_Fe(CN)_6_, 0.1% Triton X-100, and 1 mM X-Gluc. Tissues were then cleared before observation under a microscope.

**Protein subcellular localization**

GFP and YFP signals in leaves, root tips, and protoplasts were detected under a confocal laser microscope (OLYMPUS FV1000). For transient expression, observation was done at 48 h after transformation in *N. benthamiana* leaves. The root tips of the transgenic plants were incubated in 30% sucrose for cell plasmolysis.

For viewing OsBON1-eGFP signals during *Xoo* infection, roots of two-week-old seedlings of OsBON1-eGFP were inoculated with *Xoo* for 48 h as previously described (Chen *et al*., 2010). GFP fluorescence signals were detected in rice root tips. For observing OsBON1-eGFP signals during fungal blast infection, leaf sheaths from the sixth leaves of seven-leaf stage seedlings were inoculated with *M. oryzae* isolate Hoku1. Sliced-inner epidermal sections prepared from leaf sheaths inoculated with conidial suspension (1×10^5^ conidia/mL) were mounted onto a slide and observed under a confocal microscope.

**Supplementary references**

Chen, F., Gao, M.J., Miao, Y.S., Yuan, Y.X., Wang, M.Y., Li, Q., Mao, B.Z., Jiang, L.W. and He, Z.H. (2010) Plasma membrane localization and potential endocytosis of constitutively expressed XA21 proteins in transgenic rice. *Molecular Plant,* **3**, 917-926.

Ning, W., Chen, F., Mao, B., Li, Q., Liu, Z., Guo, Z. and He, Z. (2004) N-acetylchitooligosaccharides elicit rice defence responses including hypersensitive response-like cell death, oxidative burst and defence gene expression. *Physiol. Mol. Plant Pathol*, **64**, 263-271.

Tong, X., Qi, J., Zhu, X., Mao, B., Zeng, L., Wang, B., Li, Q., Zhou, G., Xu, X., Lou, Y. and He, Z. (2012) The rice hydroperoxide lyase OsHPL3 functions in defense responses by modulating the oxylipin pathway. *Plant J*, **71**, 763-775.

Wang, Z., Chen, C., Xu, Y., Jiang, R., Han, Y., Xu, Z. and Chong, K. (2004) A practical vector for efficient knockdown of gene expression in rice (*Oryza* *sativa* L.). *Plant Mol. Biol. Rep*, **22**, 409–417.

Yang, W., Gao, M., Yin, X., Liu, J., Xu, Y., Zeng, L., Li, Q., Zhang, S., Wang, J., Zhang, X. and He, Z. (2013) Control of rice embryo development, shoot apical meristem maintenance, and grain yield by a novel cytochrome p450. *Molecular plant*, **6**, 1945-1960.
